# Activated charcoal neutralization restores accurate Model for End-Stage Liver Disease and Child-Pugh scores in patients with cirrhosis on direct oral anticoagulant therapy

**DOI:** 10.1016/j.rpth.2025.103289

**Published:** 2025-12-08

**Authors:** Capucine Habay, Alix Riescher Tuczkiewicz, Imen Ben Salah, Catherine Trichet, Juliette Gay, François Durand, Pierre-Emmanuel Rautou, Olivier Roux, Emmanuelle De Raucourt

**Affiliations:** 1 Hematology laboratory, Beaujon Hospital, AP-HP – Clichy, Clichy, France; 2 Université Paris-Cité, Inserm, Centre de recherche sur l'inflammation, UMR 1149, Paris, France; 3 AP-HP, Hôpital Beaujon, Service d'Hépatologie, DMU DIGEST, Centre de Référence des Maladies Vasculaires du Foie, FILFOIE, ERN RARE-LIVER, Clichy, France

**Keywords:** activated charcoal, Child-Pugh score, factor Xa inhibitors, liver cirrhosis, model for end-stage liver disease, prothrombin time

## Abstract

**Background:**

The use of direct oral anticoagulants (DOACs) is increasingly common, including among patients with cirrhosis. These treatments interfere with coagulation tests, altering the Model for End-Stage Liver Disease (MELD) and Child-Pugh scores, which are critical for assessing disease severity and prioritizing patients on liver transplant waiting lists.

**Objective:**

To evaluate the impact of rivaroxaban and apixaban on MELD and Child-Pugh scores and assess charcoal-based neutralization.

**Methods:**

We investigated the *in vitro* impact of rivaroxaban and apixaban, at concentrations corresponding to peak plasma levels (300 ng/mL and 150 ng/mL, respectively), on the calculation of these scores. A total of 35 plasma samples from patients with cirrhosis (prothrombin level [PT%]: 13%-104%) were analyzed. INR (international normalized ratio) and PT% were measured before supplementation, after supplementation with rivaroxaban or apixaban, and after DOAC neutralization using activated charcoal (DOAC-Stop).

**Results:**

Rivaroxaban and apixaban supplementation led to an increase in INR (median: 2.81 and 0.70, respectively), resulting in a median overestimation of the MELD score by 12 and 4 points, respectively. PT% was underestimated (median: 70% for rivaroxaban and 48% for apixaban), which impacted the Child-Pugh classification in 4 and 2 patients, respectively. Neutralization of rivaroxaban and apixaban with activated charcoal resulted in INR and PT% values that were comparable to baseline measurements and remained within the analytical variability of the method.

**Conclusion:**

These findings highlight the importance of identifying patients on DOAC therapy and implementing neutralization techniques to avoid overestimating disease severity. DOAC-Stop effectively eliminates rivaroxaban- and apixaban-related interference, even in this specific population of patients with cirrhosis who sometimes have profoundly decreased PT% values. Failure to account for DOAC interference could lead to mismanagement and errors in prioritizing patients for liver transplantation.

## Essentials

• Direct oral anticoagulants interfere with hemostasis tests, even in liver disease.
• Coagulation tests were performed on cirrhotic plasma after *in vitro* rivaroxaban/apixaban supplementation.
• Rivaroxaban and apixaban distorted MELD and Child-Pugh scores, risking liver function measurement errors.
• Activated charcoal neutralized rivaroxaban/apixaban, enabling accurate and reliable test results.

## 1 Introduction

Liver insufficiency, particularly in the context of cirrhosis, leads to profound alterations in hemostasis. These alterations include decreased levels of multiple coagulation factors, reduced platelet count, and activation of endothelial cells. Consequently, patients with chronic liver disease face a dual risk of both bleeding and thrombosis [1]. Hemostasis tests, especially prothrombin level (PT%), are essential for evaluating liver dysfunction and guiding clinical management. Liver transplantation (LT) has significantly improved the prognosis of patients with decompensated cirrhosis, offering a 5-year survival rate exceeding 80%. One of the major current challenges is the accurate evaluation and timely selection of LT candidates. The shortage of available grafts, combined with increasing demand, has led to extended waiting times, which increases morbidity and mortality among patients listed for LT. Timely and appropriate referral to transplantation centers is therefore critical [2].

Although patients with cirrhosis are often excluded from clinical trials testing direct oral anticoagulants (DOACs), the use of DOACs is becoming increasingly common [3]. However, their use introduces specific challenges, particularly in the interpretation of liver function scores. The Child-Pugh and Model for End-Stage Liver Disease (MELD) scores are essential for assessing the severity of cirrhosis and determining eligibility for LT.

The Child-Pugh score, which incorporates 5 parameters (serum bilirubin, albumin, PT%, ascites, and hepatic encephalopathy), is commonly used to predict survival in patients with cirrhosis. A Child-Pugh score of C often indicates potential eligibility for LT [4]. In contrast, the MELD score is calculated using laboratory values according to the following formula: 9.57 × log(serum creatinine) + 3.78 × log(total serum bilirubin) + 11.2 × log(international normalized ratio [INR]) + 6.43. Initially developed to predict 3-month mortality in patients undergoing transjugular intrahepatic portosystemic shunt, the MELD score was subsequently validated for broader use in patients with cirrhosis [5]. It is now widely adopted to prioritize LT candidates, including in France. A MELD score >15 is generally associated with a survival benefit from LT [6]. Both scores are dynamic and require regular re-evaluation throughout the course of disease.

However, DOACs can significantly interfere with standard coagulation tests by directly inhibiting factor Xa or IIa activity. This interference affects results such as PT%, activated partial thromboplastin time (aPTT), and coagulation factor assays—including factor V—depending on the reagent, but in most cases, it often renders the results uninterpretable [[7], [8], [9]]. Most notable, DOACs may artificially elevate INR values and lower PT% results, leading to inaccurate MELD and Child-Pugh scores and potential overestimation of disease severity. These analytical artifacts can directly impact clinical decision making, highlighting the need for hemostasis laboratories to adapt their testing strategies and ensure the reliability of results in patients receiving DOAC therapy.

In this study, we aimed to assess the impact of 2 widely prescribed DOACs—rivaroxaban and apixaban—on the MELD and Child-Pugh scores. We also evaluated the potential usefulness of activated charcoal tablets to neutralize rivaroxaban and apixaban *in vitro* to restore accurate PT% and INR measurements for routine laboratory analysis.

## 2 Methods

### 2.1 Study population

We analyzed citrated plasma samples collected as part of routine care from 35 patients with cirrhosis who were hospitalized in the Hepatology Department at Beaujon Hospital (AP-HP, Paris) between May and June 2024. Patients were informed that their data would be used for research purposes. As only leftover tubes were used; no specific consent was required. Characteristics of the study population are summarized in the Table. None of the included patients were receiving anticoagulant therapy at the time of the study. Samples were centrifuged at 1500 × *g* for 15 minutes, and the plasma was subsequently frozen once before analysis.

**Table:** Patient characteristics.

Characteristics	Total *n* = 35	Rivaroxaban *n* = 18	Apixaban *n* = 17
Demographics			
Age, y	60 ± 11	58 ± 11	60 ± 11
Male, *n* (%)	23 (66)	11 (61)	12 (71)
Etiology of cirrhosis, *n* (%)			
Alcoholic	11 (31)	5 (28)	6 (35)
Metabolic	5 (14)	2 (11)	3 (51)
Alcoholic + metabolic	5 (14)	3 (17)	2 (12)
Viral infection	5 (14)	2 (11)	3 (18)
Autoimmune hepatitis	3 (9)	2 (11)	1 (6)
Other	6 (17)	4 (22)	2 (12)
Hepatocellular carcinoma, *n* (%)	7 (20)	3 (17)	4 (24)
Laboratory results			
PT, %	48 (13-104)	50 (20-104)	48 (13-83)
INR	1.63 (0.97-5.07)	1.61 (0.97-3.46)	1.63 (1.12-5.07)
FV, %	48 (20-121)	48 (20-121)	48 (20-79)
Fibrinogen, g/L	2.16 (0.51-5.41)	2.09 (0.51-5.41)	2.29 (1.01-3.95)
Creatinine, mg/dL	0.85 (0.41-4.07)	0.81 (0.48-1.27)	0.96 (0.41-4.07)
Total bilirubin, μmol/L	42 (8-503)	54 (8-344)	36 (13-503)
Albumin, g/L	25 (14-60)	26 (14-60)	25 (19-44)
**Liver score**			
MELD	13 (0-36)	15 (0-30)	13 (5-36)
MELD >15, *n* (%)	16 (46)	9 (50)	7 (41)
Child-Pugh, *n* (%)			
A	8 (23)	4 (22)	4 (24)
B	8 (23)	4 (22)	4 (24)
C	19 (54)	10 (56)	9 (53)

Data are presented as mean ± standard deviation, *n* (%), or median (minimum-maximum). FV, factor V; INR, international normalized ratio; MELD, Model for End-Stage Liver Disease; PT, prothrombin time.

### 2.2 Plasma supplementation

Plasma samples were divided into 2 groups and supplemented *in vitro*. One group was supplemented with rivaroxaban (*n* = 18; SML2844, Sigma-Aldrich) at 300 ng/mL, corresponding to peak plasma levels observed with a 20 mg once-daily dose. The other group was supplemented with apixaban (*n* = 17; SML3285, Sigma-Aldrich) at 150 ng/mL, reflecting peak plasma levels associated with a 5 mg twice-daily dose [10]. Rivaroxaban and apixaban powders were dissolved in dimethyl sulfoxide (2 mg/mL stock solution), serially diluted, and finally added to plasma samples at a 1:100 dilution to achieve the target concentrations. The supplemented plasma concentrations of rivaroxaban and apixaban were verified by performing tests of drug-specific anti-Xa activity (STA-Liquid Anti-Xa; Stago).

### 2.3 DOAC removal

To remove anti-Xa DOACs activity from plasma, activated charcoal (DOAC-Stop; Cryopep) was used in accordance with the manufacturer’s instructions. After DOAC-Stop treatment, a heparin-calibrated anti-Xa activity assay was performed to confirm the absence of residual anti-Xa activity. The safety of DOAC-Stop and its lack of interference in samples from patients not receiving anticoagulant therapy have been previously demonstrated [11].

### 2.4 PT% and INR determination

PT% and INR were measured using an automated coagulation analyzer (STAR; Stago), using the manufacturer’s reagents (STA-Néoptimal 20; Stago) and protocols. PT% is expressed as a percentage, derived from a calibration curve (Thivolle plot) that expresses clotting performance relative to that of normal plasma. Measurements were performed on baseline plasma samples, after *in vitro* supplementation with rivaroxaban or apixaban, and following DOAC neutralization with DOAC-Stop.

### 2.5 MELD and Child-Pugh score

MELD and Child-Pugh scores were calculated using the values obtained before and after rivaroxaban or apixaban supplementation and after charcoal neutralization.

### 2.6 Statistical analysis

Quantitative variables are expressed as medians (minimum-maximum) and qualitative variables as counts and percentages (n [%]). The Mann–Whitney U-test was used to compare continuous variables. Spearman’s correlation coefficient was applied to assess associations between variables. Comparison between initial and post-neutralization INR and PT% values were performed using the Wilcoxon signed-rank test for paired samples. All statistical analyses and graphical representations were conducted using GraphPad Prism version 8.0 (GraphPad Software), with statistical significance set at *P* < .05.

## 3 Results and discussion

### 3.1 Impact on MELD score

**Figure 1:**
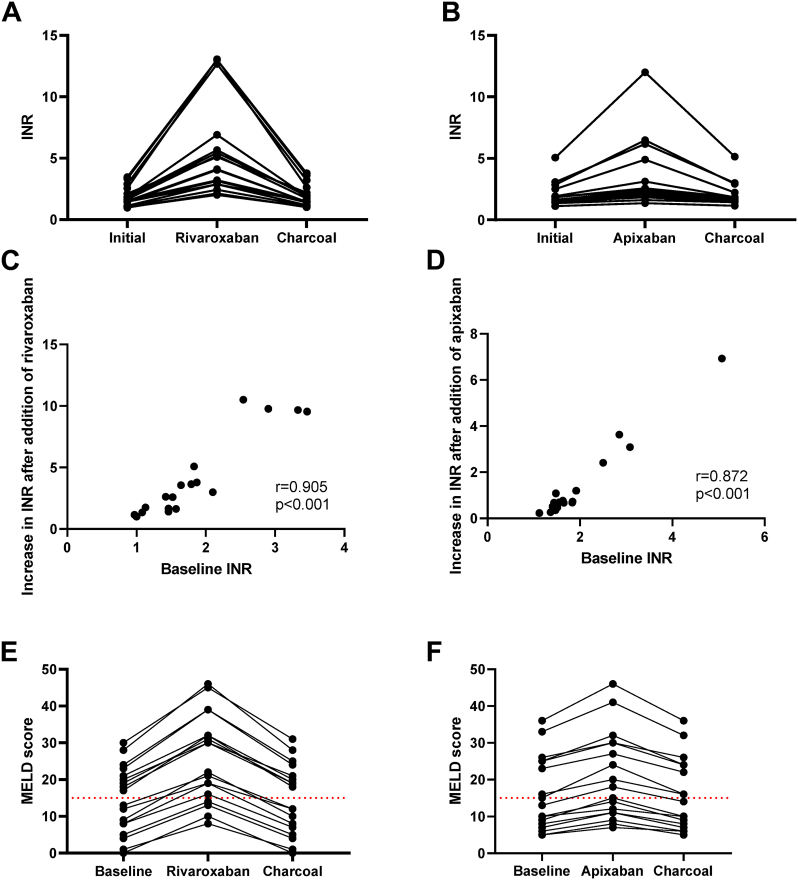
Impact of rivaroxaban and apixaban supplementation on MELD score. A, C, E: Impact of rivaroxaban supplementation. B, D, F: Impact of apixaban supplementation. A, B: Evolution of international normalized ratio (INR) after rivaroxaban or apixaban supplementation and neutralization using activated charcoal. C, D: Correlation of baseline INR values to the absolute increase in the INR values after the addition of rivaroxaban or apixaban. E, F: Impact of rivaroxaban or apixaban supplementation and charcoal neutralization on MELD score calculation. MELD, Model for End-Stage Liver Disease.

*In vitro* supplementation of plasma with rivaroxaban resulted in a significant overestimation of INR values, with median increase of 2.80 (1.01-10.51), leading to a corresponding increase in MELD score from a baseline median of 18 (8-30) to 30 (16-46) after supplementation (Figure 1A, E). This represented a median overestimation of 11.5 points (range: 8-18). Notably, among the 9 patients with a baseline MELD score ≤15, 5 exceeded this clinical threshold after rivaroxaban supplementation.

Similarly, apixaban supplementation led to INR overestimation, although to a lesser extent, with a median increase of 0.70 (0.23-6.93). The MELD score increased from a median of 13 (5-36) to 18 (7-46) (Figure 1B, F), corresponding to a median increase of 4 points (2-10). Of the 10 patients whose initial MELD score was ≤15, 2 crossed this threshold after apixaban supplementation.

The overestimation of INR—and consequently of the MELD score—was significantly greater with rivaroxaban than with apixaban (both *P* < .001). Furthermore, the extent of INR overestimation was positively correlated with the baseline INR; the higher the initial INR, the greater the overestimation induced by rivaroxaban or apixaban supplementation (*r* = 0.905, *P* < .001 and *r* = 0.872, *P* < .001 respectively) (Figure 1C, D).

These findings are consistent with data reported by Lisman et al. [12], who demonstrated that *in vitro* supplementation of 20 plasma samples from liver transplant candidates with various DOACs (dabigatran, rivaroxaban, apixaban, and edoxaban) resulted in MELD score increases ranging from 3 to 10 points. This highlights the clinical significance of DOAC interference during INR measurement, as it can alter eligibility criteria for LT. In many settings, a MELD score >15 serves as a key threshold for transplant listing. In our cohort, the artificial MELD score inflation caused by DOAC presence would have incorrectly qualified several patients for transplantation listing.

### 3.2 Impact on Child-Pugh score

**Figure 2:**
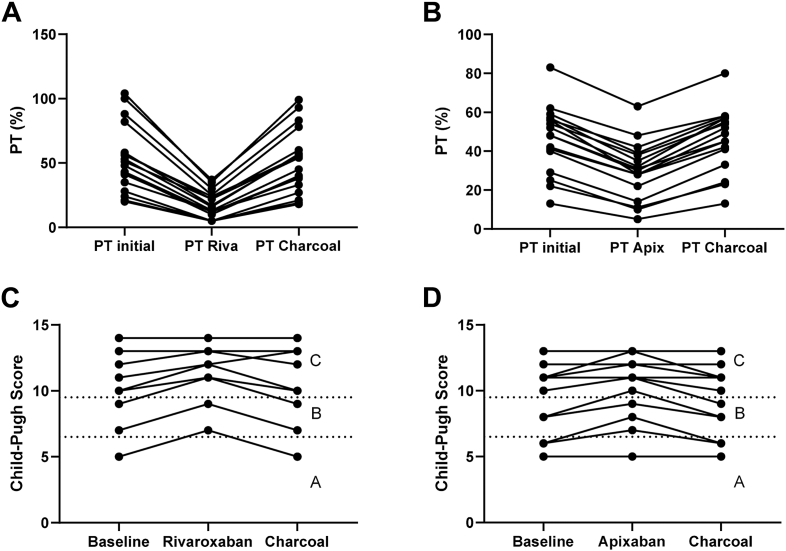
Impact of rivaroxaban and apixaban supplementation on Child-Pugh score. A, C: Impact of rivaroxaban (Riva) supplementation. B, D: Impact of apixaban (Apix) supplementation. A, B: Variation in prothrombin level (PT%) after rivaroxaban or apixaban supplementation and subsequent neutralization using activated charcoal. C, D: Effect of rivaroxaban and apixaban supplementation and charcoal neutralization on the Child-Pugh score.

In the rivaroxaban group, *in vitro* supplementation underestimated PT%, with values decreased from a baseline of 50% (20%-104%) to 15% (5%-37%), representing a median decrease of 70% (57%-82%). This substantial reduction resulted in an overestimation of the Child-Pugh score, with potential clinical consequences. Four patients were reclassified from Child-Pugh A to Child-Pugh B and 1 patient from Child-Pugh B to Child-Pugh C (Figure 2A, C).

In the apixaban group, supplementation caused a median PT% underestimation of 38% (23%-62%), with values falling from 48% (13%-83%) to 30% (5%-63%). This also impacted Child-Pugh classification. Two patients were reclassified from Child-Pugh A to B, and one from Child-Pugh B to C (Figure 2B, D).

These observations confirm that the presence of apixaban and rivaroxaban leads to clinically significant interference with coagulation parameters, directly affecting liver disease severity assessment. Notably, rivaroxaban appeared to have a more pronounced impact on PT% than apixaban (*P* < .001). If unrecognized, such interferences could lead to inappropriate clinical decision making.

### 3.3 *In vitro* evaluation of charcoal to ensure accurate PT% and INR in patients with cirrhosis on rivaroxaban or apixaban

In the rivaroxaban group, the baseline INR was 1.61 (0.97-3.46) and remained at 1.61 (1.01-3.78) after supplementation followed by charcoal neutralization (*P* = .01), with no individual deviation greater than 0.32 points. Similarly, PT% remained stable, with values of 49.5% (20%-104%) before and 49.5% (18%-99%) after neutralization (*P* = .02), and no deviation exceeded 7% (Figures 1A and 2A).

In the apixaban group, the baseline INR was 1.63 (1.12-5.07) and remained unchanged after neutralization at 1.63 (1.15-5.14) (*P* = .54), with no individual deviation greater than 0.28 points. PT% also remained stable, with an initial value of 48% (13%-83%) and a posttreatment value of 49% (13%-80%) (*P* = 1.00), with no deviation greater than 7% (Figures 1B and 2B).

Only the INR and PT% values in the apixaban group showed no statistically significant difference between the baseline and post-neutralization measurements. However, in the rivaroxaban group, although statistical differences were observed, the variation in INR and PT% values before and after neutralization remained within acceptable analytical limits. Specifically, the percentage difference never exceeded 2.8 times the method’s coefficient of variation—7.9% for low values (<69%) and 5.6% for high values (>68%)—thus falling within the expected measurement uncertainty [13]. These findings demonstrate that rivaroxaban and apixaban adsorption using activated charcoal tablets effectively eliminates drug-related interferences, even in this specific population of patients with cirrhosis who may present with markedly low PT% values. This approach enables accurate PT% and INR measurements, ensuring reliable calculation of MELD and Child-Pugh scores. A limitation of our study is that only a single PT reagent was evaluated; nevertheless, it is important to note that most PT reagents exhibit sensitivity to the presence of DOACs, suggesting that our findings may be broadly applicable [9].

An alternative approach would be to measure PT% and INR after temporary discontinuation of DOAC therapy. However, this is not always feasible, particularly in emergency settings. Moreover, because DOACs are metabolized by both the liver and kidneys, their clearance may be impaired in patients with cirrhosis and concomitant renal dysfunction, leading to prolonged drug persistence and interference with coagulation testing.

Although activated charcoal offers a simple, effective, and low-cost solution to neutralize DOACs in coagulation assays and minimize their impact on MELD and Child-Pugh scores, its success depends on the laboratory being informed of the patient’s anticoagulant therapy. In our routine practice, this information is frequently missing at the time of sample receipt. It is also common for emergency physicians to be unaware of the patient’s anticoagulant status, either because the patient omits it or is unable to communicate it.

To address this issue, our laboratory implemented DOAC screening for emergency department patients with unexplained reduced PT% and/or prolonged aPTT when no anticoagulant therapy was indicated. Heparin-calibrated anti-Xa activity and thrombin time are measured. Presence of a DOAC is suspected only when anti-Xa activity is markedly elevated with a normal thrombin time or when thrombin time is prolonged with no detectable anti-Xa activity; in other cases, heparin intake is considered more likely. When a DOAC is suspected, we contact the attending physician to confirm the patient’s anticoagulant therapy. If this information is unavailable, the physician typically verifies it directly with the patient. In cases in which a DOAC is present, PT and aPTT results are not reported. Depending on the clinical context (such as urgent surgical procedures, reassessment for LT eligibility), coagulation tests are performed after charcoal-based neutralization to ensure reliable results and avoid erroneous clinical decisions. Only post-neutralization results are reported, specifying that they correspond to *in vitro* DOAC neutralization.

Although a formal economic analysis to assess costs was not performed, it would certainly be valuable. Over a 15-day period in our laboratory, 41 of 481 hemostasis panels (9%) required these assays, identifying DOAC intake in 19 cases (46%), whereas 20 cases (49%) were negative. In these 19 cases, this procedure prevented the reporting of potentially misleading results. During this period, no clinical situation ultimately required reporting results after charcoal-based neutralization. It is noteworthy that the development of DOAC-insensitive reagents would be of interest. Our study extends the findings previously reported by Lisman et al. [12] by specifically evaluating the impact of apixaban and rivaroxaban on the Child-Pugh score and by assessing the utility of charcoal-based DOAC neutralization in this particular population of patients with cirrhosis.

In conclusion, in patients with cirrhosis receiving rivaroxaban or apixaban for thromboembolic events or atrial fibrillation, we demonstrated that there is a significant risk of MELD and Child-Pugh score overestimation, which can lead to an inaccurate assessment of disease severity. We also showed that rivaroxaban and apixaban neutralization using activated charcoal allows for reliable restoration of these severity scores. Therefore, it is essential to raise clinician awareness of this risk, ensure effective communication, and implement targeted strategies within hemostasis laboratories to identify patients on DOAC therapy. When appropriate, charcoal-based neutralization should be proposed to guarantee accurate results and informed clinical decision making.

## References

[1] Tripodi A., Anstee Q.M., Sogaard K.K., Primignani M., Valla D.C. (2011). Hypercoagulability in cirrhosis: causes and consequences. J Thromb Haemost.

[2] Durand F., Belghiti J. (2005). Transplantation hépatique chez l'adulte [Liver transplantation in adults] [in French]. Med Sci (Paris).

[3] European Association for the Study of the Liver (2022). EASL Clinical Practice Guidelines on prevention and management of bleeding and thrombosis in patients with cirrhosis. J Hepatol.

[4] Gex L., Bernard C., Spahr L. (2010). Scores en hépatologie: Child-Pugh, MELD et Maddrey [Child-Pugh, MELD and Maddrey scores] [in French]. Rev Med Suisse.

[5] Kamath P.S., Wiesner R.H., Malinchoc M., Kremers W., Therneau T.M., Kosberg C.L. (2001). A model to predict survival in patients with end-stage liver disease. Hepatology.

[6] Merion R.M., Schaubel D.E., Dykstra D.M., Freeman R.B., Port F.K., Wolfe R.A. (2005). The survival benefit of liver transplantation. Am J Transplant.

[7] Mani H. (2014). Interpretation of coagulation test results under direct oral anticoagulants. Int J Lab Hematol.

[8] Exner T., Dangol M., Favaloro E.J. (2024). Simplified method for removing direct oral anticoagulant interference in mechanical coagulation test systems-a proof of concept. J Clin Med.

[9] Samama M.M., Martinoli J.L., LeFlem L., Guinet C., Plu-Bureau G., Depasse F. (2010). Assessment of laboratory assays to measure rivaroxaban--an oral, direct factor Xa inhibitor. Thromb Haemost.

[10] Douxfils J., Adcock D.M., Bates S.M., Favaloro E.J., Gouin-Thibault I., Guillermo C. (2021). 2021 update of the International Council for Standardization in Haematology recommendations for laboratory measurement of direct oral anticoagulants. Thromb Haemost.

[11] Platton S., Hunt C. (2019). Influence of DOAC Stop on coagulation assays in samples from patients on rivaroxaban or apixaban. Int J Lab Hematol.

[12] Lisman T., Bernal W., Adelmeijer J., Kamphuisen P.W., Bos S., Porte R.J. (2023). Clinically relevant increases in the international normalized ratio and model of end-stage liver disease score by therapeutic doses of direct oral anticoagulants in patients with cirrhosis. Res Pract Thromb Haemost.

[13] Flaujac C., Marion S., Roussi J., Cambus J.P., Stepanian A. (2014). Normes d’acceptabilité en hémostase [Acceptability standards in hemostasis]. Groupe d'Etudes sur l'Hemostase et la Thrombose (GEHT).

